# Adult-Onset Multifocal Cutaneous Myofibromas: A Case Report of a Rare Entity

**DOI:** 10.7759/cureus.52438

**Published:** 2024-01-17

**Authors:** Alex K Miller, Nassim Lashkari, Ashbita Pokharel, Drew D Moore

**Affiliations:** 1 Orthopedic Surgery, Corewell Health William Beaumont University Hospital, Royal Oak, USA; 2 Pathology, Corewell Health William Beaumont University Hospital, Royal Oak, USA

**Keywords:** smooth-muscle actin, multifocal infantile myofibroma, infantile myofibroma, cutaneous myofibroma, benign, multifocal, myofibromatosis, adult-onset, myofibromas

## Abstract

Myofibromas are observed in both infantile and adult presentations, with key differences in the number and severity of lesions between these two groups. Infantile presentations encompass both indolent, isolated cutaneous lesions, as well as aggressive, multicentric presentations with visceral involvement. Adult myofibromas appear to be characterized by a single isolated cutaneous lesion, generally asymptomatic and following a benign clinical course. The occurrence of adult multifocal myofibromas has not yet been described in the literature. Here, we report a case of a 57-year-old female who presented with two minimally symptomatic soft tissue lesions on her right leg, with the pathologic findings of each lesion consistent with a cutaneous myofibroma. This case report describes a rare presentation of adult-onset multifocal cutaneous myofibromas.

## Introduction

Myofibromas represent a localized proliferation of myofibroblasts and are the most common fibrous tumor of infancy [1]. Typically seen in the first two years of life, infantile myofibromatosis has been reported to occur in one out of 150,000 to 400,000 live births [2,3]. Solitary myofibromas have also been observed in adult patients, though the incidence within this population has not been well documented. In the infantile presentation, these tumors typically present as benign solitary lesions of the skin, subcutaneous tissue, or muscle. However, some patients are affected by a more aggressive, multicentric presentation, with lesions involving soft tissue, bone, and possibly viscera [4]. Adult myofibromas are typically characterized by a solitary lesion, located in the dermis, with benign behavior [5]. No consistently reported environmental risk factors have been identified for myofibromatosis; however, various genetic associations have been reported in familial forms of infantile myofibromatosis, including germline mutations in platelet-derived growth factor receptor beta (PDGFRB) [6].

A presentation of multicentric adult myofibromas has not previously been described, with current literature limited to adolescents presenting with multiple extremity lesions after known infantile myofibromatosis [1,6,7,8]. We present a case of an adult-onset multifocal cutaneous myofibromas, which, to our knowledge, is the only reported case in the literature to date.

## Case presentation

A 57-year-old female was referred to the orthopedic oncology clinic with a four-year history of two soft tissue masses, located on her right lateral knee and right anterolateral ankle. These masses initially appeared very small but demonstrated growth over the year before presentation; at the time of her visit, each mass was approximately 4 cm in size. Symptoms attributable to these lesions included vague ankle pain and mild dorsal foot discomfort with prolonged standing. She denied a previous history of cancer or similar findings elsewhere in her body. There were no new systemic symptoms, including fevers or chills. She reported no unintentional weight loss. She was noted to be a current everyday smoker at the time of the initial presentation. She did not have a history of occupational exposures, prior chemotherapy, or prior radiation treatments. 

Upon examination, the knee and ankle masses were ovoid and noted to be firm but mobile with direct palpation. Mobility of the knee mass appeared to demonstrate mild adherence to the overlying skin; this was not observed at the ankle mass. Some overlying erythema was present in the skin overlying the lateral knee mass; no skin changes were observed over the ankle mass. A Tinel’s sign (radiating paresthesias with direct tapping of the lesion, indicating neural/perineural involvement) was not present over either site.

**Figure 1:**
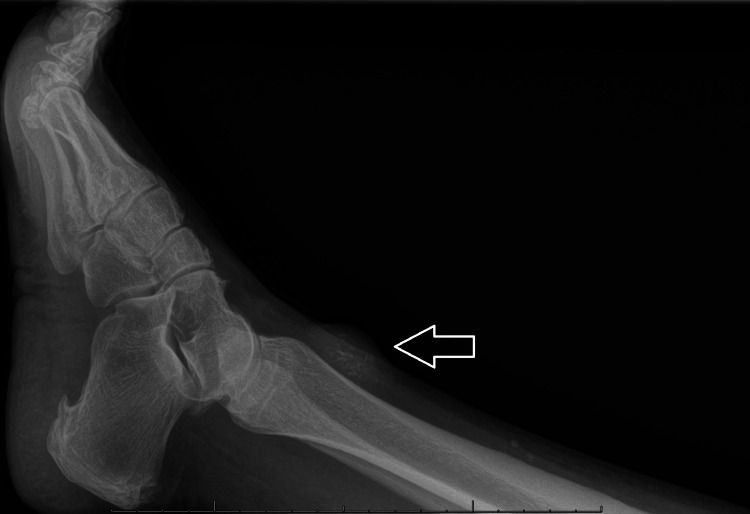
Lateral radiograph of right ankle demonstrating soft tissue mass with calcifications (arrow).

Radiographs demonstrated mild calcifications in the lateral ankle mass and subtle calcifications in the knee mass, as illustrated in the ankle mass image (Figure 1).

**Figure 2:**
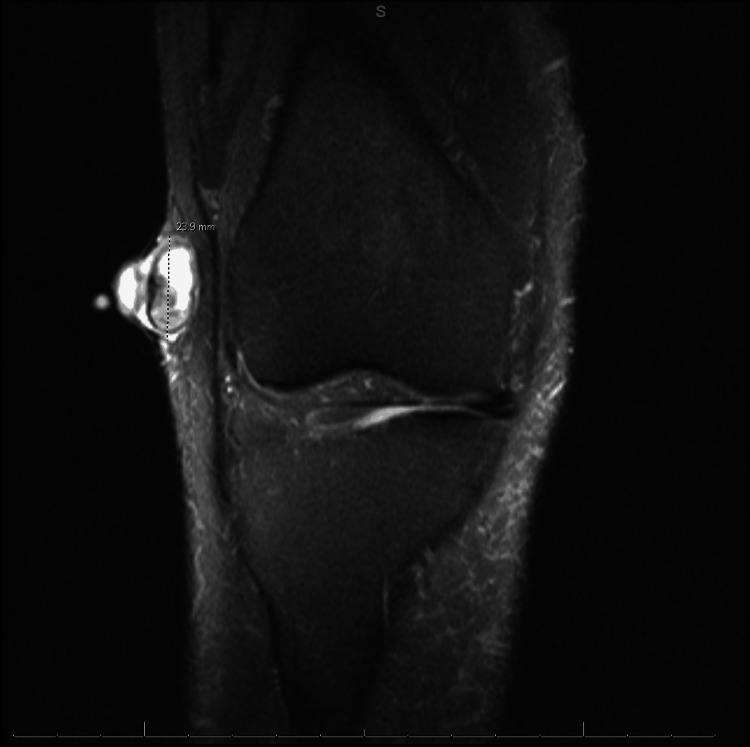
Coronal MRI (proton density-weighted, fat-suppressed, turbo spin echo) demonstrating the structure of the lesion along the lateral aspect of the right knee. MRI, magnetic resonance imaging

The MRI of the knee revealed a significant lesion within the subcutaneous soft tissues, measuring approximately 2.2 cm x 2.2 cm x 2.4 cm. The lesion exhibited a predominantly bright T2 signal, as depicted in the right knee coronal MRI (Figure 2).

**Figure 3:**
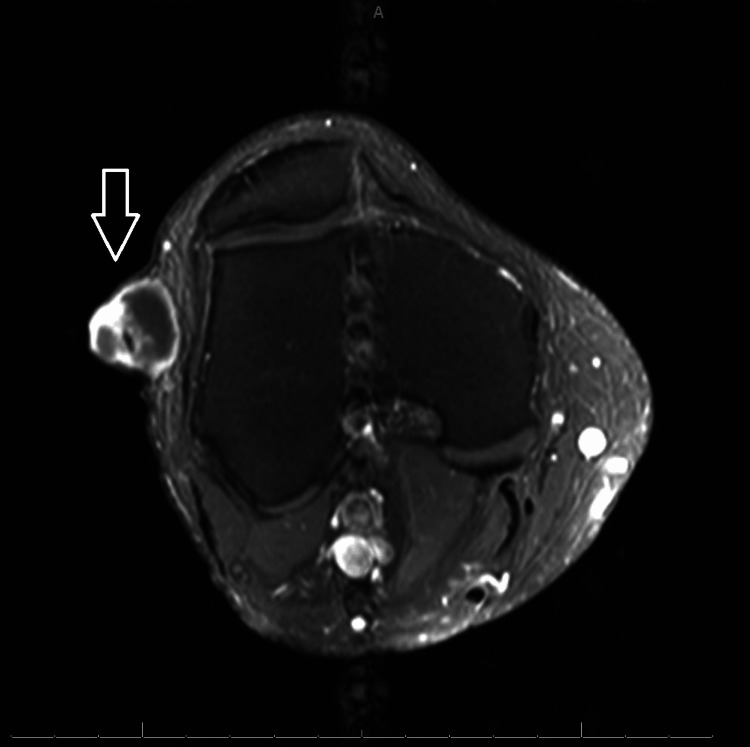
Axial MRI of the right knee (T1-weighted, post-contrast) demonstrating peripheral contrast enhancement along the laterally located lesion (arrow). MRI, magnetic resonance imaging

Additionally, some areas of hypo- and hyperintense T1 signals were present. Peripheral enhancement was noted with gadolinium-based contrast, as depicted in the contrast-enhanced axial right knee MRI (Figure 3).

MRI of the ankle demonstrated a 2.2 cm x 2.0 cm x 1.8 cm lesion contained within the subcutaneous tissues. This mass also demonstrated a well-circumscribed appearance, with mainly T2-intense signal, surrounded by rings of T1 hypointense signal. Given the nonspecific findings on MRI, the presence of multiple lesions, and slow growth before presentation, excision for pathologic diagnosis was recommended.

The procedure was performed by an orthopedic oncologist. The knee mass was excised en bloc, including the overlying skin that appeared contiguous with the mass. Careful dissection proceeded circumferentially around the lesion, with margins of normal subcutaneous fat. A 4 cm x 3 cm specimen was removed in one piece, without evidence of tumor spill or contamination. The ankle mass appeared slightly deeper and without skin involvement; a longitudinal incision was made in the skin overlying the lesion, with medial and lateral flaps raised with electrocautery. A branch of the superficial peroneal nerve was found to be adherent to tissues adjacent to the mass. The mass was dissected free while maintaining a margin of surrounding subcutaneous fat. A 3 cm x 3 cm specimen was delivered from the field in one contiguous piece.

**Figure 4:**
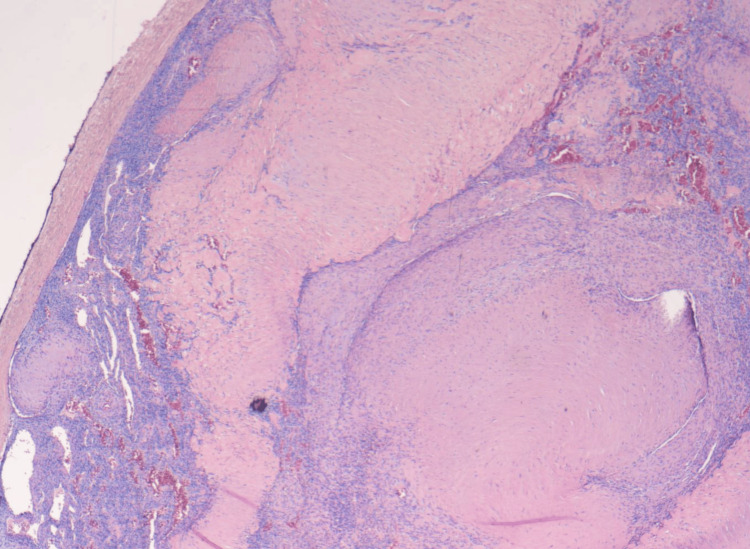
Hematoxylin and eosin-stained specimen at low magnification (4x), illustrating nodular collections of spindle-like myofibroblasts.

The gross pathology of the right knee mass revealed a firm, white-tan, glistening nodule measuring 2.0 cm x 2.0 cm x 1.6 cm. The nodule exhibited focal gritty areas and contained a central cavity filled with clear, white fluid. The remaining tissue had a lobulated, tan-yellow appearance. The skin included in the knee specimen abutted the lesion without invading the overlying skin. The ankle mass had a similar gross appearance, with white-tan rubbery surfaces and focal areas of hemorrhage and calcification. The overall size of the ankle lesion was 2.7 cm x 2.5 cm x 2.0 cm. A central cavity of 0.7 cm in diameter and devoid of contents was noted. The microscopic appearance revealed nodular proliferations of eosinophilic, fusiform myofibroblasts surrounded by less differentiated pericytes, as shown in the histopathologic microphotograph (Figure 4). No tumor involvement of the margins was observed for either specimen.

Immunohistochemistry showed a positive result for smooth muscle actin immunostain and negative results for CD34 and desmin. A diagnosis of myofibroma was established for both masses.

The patient underwent follow-up appointments at two weeks, one month, and three months postoperatively to monitor incisional healing. During these visits, she was informed that recurrence may still be possible, despite the curative intent of the resection. Before the preparation of this report, she was contacted by telephone approximately three years after the surgery. At this time, she confirmed no local recurrence or other new lesions and offered her consent for publication of this case report.

## Discussion

To our knowledge, this case represents the first reported incidence of adult-onset multifocal myofibromas. Clinically, myofibromas have been described as a solitary nodule in the subcuticular and dermal layers of the skin with the presence of disease reported to be nearly twice as common in males compared with females [2,9,10]. The majority of myofibromas occur in early infancy, with the solitary subtype being more common than the multicentric subtype among infants [11]. Solitary nodules have been reported to occur most commonly in the head and neck region, with the trunk being the second most common, followed by the lower and upper extremities, respectively [2,12,13]. A small number of cases have been reported to be inherited in an autosomal dominant manner [14,15,16]. The clinical course of lesions within the bone and soft tissue is benign, with the majority of lesions regressing spontaneously and rare instances of local recurrence [14].

In the histologic description of myofibromas, the microscopic appearance of the cells is commonly described as nodular proliferation, with plump spindle cells containing eosinophilic cytoplasm [14,17]. The characteristics of the central and peripheral areas of the lesions have consistent pathological differences, with the peripheral regions consisting of monomorphic spindle fibroblastic cells and the central regions consisting of richly vascular nodules often mimicking the appearance of a hemangiopericytoma [1,18,19]. In adults, this arrangement of cells in central and peripheral regions may be random or even reversed [20]. In some cases, chronic inflammatory cells, focal hemorrhage with cystic degeneration, or coagulative necrosis may be present, which may be mistaken for sarcoma [14]. Immunohistochemistry and genetic assays can be used to assist in the differential diagnosis. The presence of PDGFRB mutations has additionally been reported in solitary adult lesions [21]. The most useful histologic finding for recognizing myofibromatosis is the identification of peripheral myoid-appearing cells, assisted by positive immunohistochemistry staining for muscle-specific actin and smooth muscle actin, along with the absence of desmin [2,20,22].

Though imaging findings are insufficient for solidifying a diagnosis, the radiographic characteristics of MRI are largely consistent. Myofibromas are noted to exhibit an isointense appearance to muscle on non-contrast T1-weighted images and a hyperintense appearance on T2-weighted images [23]. Also commonly observed is central necrosis and peripheral enhancement, often with calcification [24]. In a case series involving five MRIs in infants with non-cranial myofibromatosis, three out of five masses exhibited a center with a high T2 signal, and all five masses showed peripheral enhancement with gadolinium contrast [25]. Despite the consistencies in imaging appearance among myofibromas, these radiographic features are nonspecific and require histologic evaluation to confirm the diagnosis [23].

The incidence of adult myofibromas is rare, with a review of the current literature limited to the presence of multiple extremity lesions in adolescents after known infantile myofibromatosis. In a case series of 61 infants and adolescents described by Chung and Enzinger, 16 cases of multicentric myofibromas were described [1]. Among these patients, there was further distinction by the presence of visceral involvement. In the long-term follow-up, five out of 12 cases with multicentric involvement showed spontaneous regression. However, one patient exhibited new growth at the age of 15 years, and another patient developed five other nodules within 3.5 years after the initial surgical resection. Another case of recurrence in adulthood has been reported in a patient who initially presented with a superficial scalp lesion at two years old [7]. This patient was observed to have additional primary myofibromas in the right mandibular vestibule at age 9, the right temple at age 12, and the left mandibular vestibule at age 23. Foss and Ellis documented two cases of multifocal myofibromas from a registry of the Armed Forces Institute of Pathology. Both patients presented with intraoral bone lesions and multiple subcutaneous tumors; however, each patient also had a known history of previous infantile myofibromatosis [8].

## Conclusions

The incidence of both infantile and adult myofibromas is rare, with the presentation of multifocal cutaneous lesions in an adult not previously reported in the literature. In the infantile population, solitary myofibromas have an excellent prognosis. Similarly, the presentation of a solitary myofibroma in an adult is regarded as benign, with surgical resection recommended for those lesions with local mass effect or progressive growth. In an infant, multicentric and generalized myofibromatosis requires close follow-up for progressive disease. Until further cases emerge, the prognosis of adult myofibromas cannot be fully defined, though it appears similar to that of a solitary myofibroma. Further investigations are needed to understand the biological pathways underlying the varying temporal presentations of myofibromatosis, including the role of PDGFRB and potential familial patterns of inheritance.
